# Applying Evidence-Based Violence Prevention Strategies to Elder Abuse in Public Health

**DOI:** 10.1093/geroni/igaa057.148

**Published:** 2020-12-16

**Authors:** Carolyn Ham, Cory Bolkan

**Affiliations:** 1 Washington State Department of Health, Olympia, Washington, United States; 2 Washington State University, Vancouver, Washington, United States

## Abstract

Elder abuse is a growing problem with significant public health implications. Because elder abuse shares root causes with other types of violence (e.g., suicidal behavior, intimate partner violence), awareness of elder abuse as a violence prevention priority is rising among public health professionals. Major limitations, however, affect delivery of effective population-level primary prevention for elder abuse, necessitating increased community partnerships. In Washington State, the Department of Health’s Injury and Violence Prevention Section and the Department of Social and Health Services Adult Protective Services Division are leveraging existing strategies to increase identification and reporting of potential elder abuse from falls and injury prevention partners (i.e., opioids, suicide). We describe: (1) challenges and opportunities in creating unique cross-program collaborations, (2) the combined education and outreach efforts of this partnership, and (3) strategies for sustained collaboration. Additionally, we share results of a scoping literature review on evidence-based violence prevention strategies applicable to elder abuse between 2015 – 2019. In the Pubmed and Academic Search Complete databases, the following terms were searched: elder abuse prevention, primary prevention, shared risk and protective factors. Only six articles were identified that addressed primary prevention efforts. Researchers note that primary prevention of elder abuse is poorly understood and challenges exist in applying methods from other types of violence. Education for key community members on identification of abuse is a promising intervention targeting shared risk and protective factors for public health to pursue. Cross-sector community partnerships and rigorous evaluation of primary prevention approaches are needed.

